# The Multifaceted Role of Alpha-Lipoic Acid in Cancer Prevention, Occurrence, and Treatment

**DOI:** 10.3390/antiox13080897

**Published:** 2024-07-25

**Authors:** Shuai Yan, Jiajie Lu, Bingqing Chen, Liuxia Yuan, Lin Chen, Linglin Ju, Weihua Cai, Jinzhu Wu

**Affiliations:** 1 Medical School, Nantong University, Nantong 226300, China; ys867218209@icloud.com (S.Y.); 15862823913@163.com (J.L.); cbq1999@163.com (B.C.); 2 Institute of Liver Diseases, Affiliated Nantong Hospital 3 of Nantong University, Nantong 226300, China; yuan957@ntu.edu.cn (L.Y.); orange303@ntu.edu.cn (L.C.); 5300958@ntu.edu.cn (L.J.); 3 Department of Hepatobiliary Surgery, Affiliated Nantong Hospital 3 of Nantong University, Nantong 226300, China; cwhntsy@163.com

**Keywords:** alpha-lipoic acid, cancer, antioxidants, reactive oxygen species, oxidative stress, nanomedicine

## Abstract

Alpha-lipoic acid (ALA) is a naturally occurring compound synthesized by mitochondria and widely distributed in both animal and plant tissues. It primarily influences cellular metabolism and oxidative stress networks through its antioxidant properties and is an important drug for treating metabolic diseases associated with oxidative damage. Nevertheless, research indicates that the mechanism by which ALA affects cancer cells is distinct from that observed in normal cells, exhibiting pro-oxidative properties. Therefore, this review aims to describe the main chemical and biological functions of ALA in the cancer environment, including its mechanisms and effects in tumor prevention and anticancer activity, as well as its role as an adjunctive drug in cancer therapy. We specifically focus on the interactions between ALA and various carcinogenic and anti-carcinogenic pathways and discuss ALA’s pro-oxidative capabilities in the unique redox environment of cancer cells. Additionally, we elaborate on ALA’s roles in nanomedicine, hypoxia-inducible factors, and cancer stem cell research, proposing hypotheses and potential explanations for currently unresolved issues.

## 1. Introduction

**Figure 1:**
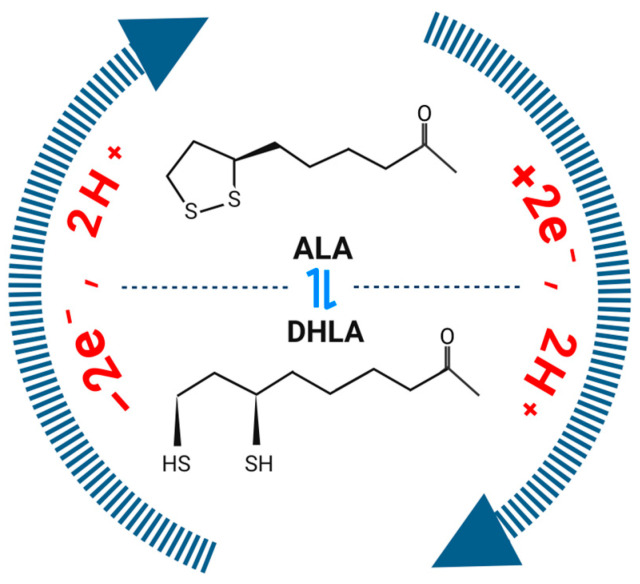
Schematic diagram of the chemical structures of ALA and its reduced form DHLA. In different redox environments, these structures can be interconverted. Created with BioRender.com (accessed on 22 July 2024).

Alpha-lipoic acid (1,2-dithiolane-3-pentanoic acid, ALA) and its reduced form, dihydrolipoic acid (DHLA, Figure 1), are natural antioxidants that are widely distributed in both plants and animals [1,2]. Since its discovery in 1937, ALA has garnered increasing attention due to its simple structure and complex functions [3]. In humans, ALA primarily originates from two sources: endogenous de novo synthesis and exogenous intake [4,5,6]. As a key compound, ALA is an essential cofactor for the activity of four mitochondrial enzyme complexes involved in energy metabolism: pyruvate dehydrogenase (PDHC), the glycine cleavage system (GCS), branched-chain 2-ketoacid dehydrogenase (BCKDH), and 2-ketoglutarate dehydrogenase (2-KGDH) [6]. As a mitochondrial nutrient (MN), ALA not only is a crucial component of glucose metabolism and ATP generation but also has antioxidant functions, protecting normal cells from the damaging effects of free radicals (FRs), also known as reactive oxygen species (ROS) [7]. Additionally, ALA acts as a metal chelator in certain metal ion accumulation phenomena, and its amphipathic characteristics make it a suitable chelating agent in nanotherapy, aiding drugs in achieving cellular selectivity and safety [8,9]. Given these features, ALA has been found to be applicable in the treatment of chronic diseases related to inflammation and oxidative stress. In recent years, the anticancer effects of ALA have also been demonstrated, including the promotion of oxidation, inhibition of pro-cancer pathways, and activation of tumor suppressor genes, and is posited to play roles in various stages of cancer development [10].

Herein, we aim to review the primary chemical and biological functions of ALA in tumor prevention and anticancer activity and its role as an adjuvant in cancer therapy. We focus on its interactions with multiple pro-cancer and anticancer pathways, proliferation, apoptosis, epithelial–mesenchymal transition (EMT), and cancer stem cells (CSCs) by summarizing previous literature. Additionally, we explore the role of ALA as a chelating agent in cutting-edge nanomedicine.

## 2. ALA and Tumor Prevention

### 2.1. Role in Maintaining Redox Homeostasis in the Body

**Figure 2:**
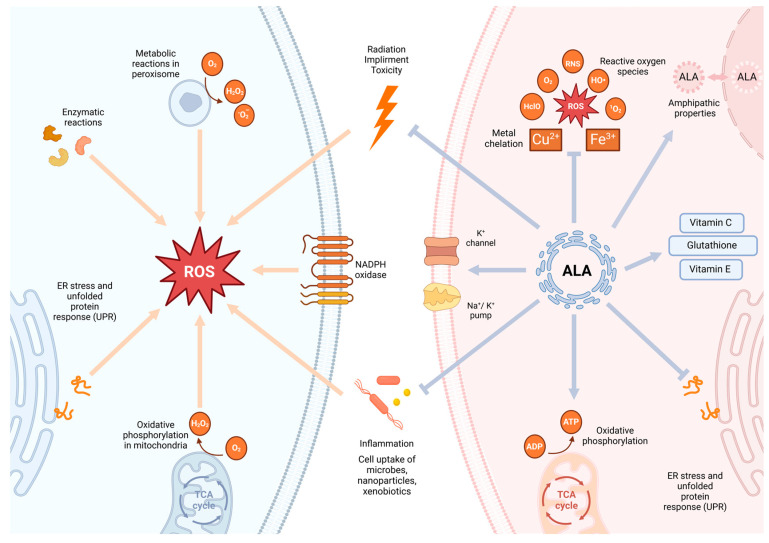
Sources of ROS and the antioxidative effects of ALA. Sources of ROS: The blue section on the left side of the figure outlines the extracellular and intracellular sources of ROS production. Extracellular sources of ROS include environmental pollutants, radiation, toxic stimuli, microbial infections, and the inflammation they induce. Intracellularly, both normal and abnormal cellular activities can induce ROS production, including mitochondrial oxidative phosphorylation, ER stress and the UPR, enzymatic reactions, peroxisome metabolism, and NADPH oxidase activity. Antioxidative effects of ALA: The red section on the right side of the figure summarizes the role of ALA as a multifunctional antioxidant in protecting cells from excessive ROS damage. ALA can directly quench various reactive oxygen species, including ROS, RNS, HO^•^, H_Cl_O, and ^1^O_2_. It also provides indirect antioxidant protection through metal chelation (e.g., Cu^2+^ and Fe^3+^) and the regeneration of certain endogenous antioxidants (e.g., vitamin E, vitamin C, and glutathione). Additionally, ALA leverages its amphiphilic properties to distribute in both hydrophilic environments (e.g., plasma) and lipophilic environments (e.g., cell membranes). Finally, ALA’s antioxidative action effectively protects mitochondria, ensuring efficient ATP production and thereby maintaining the proper function of the sodium–potassium pump [24]. (Arrows indicate promotion, lines indicate inhibition). Created with BioRender.com (accessed on 22 July 2024). RNS: reactive nitrogen species.

**Figure 3:**
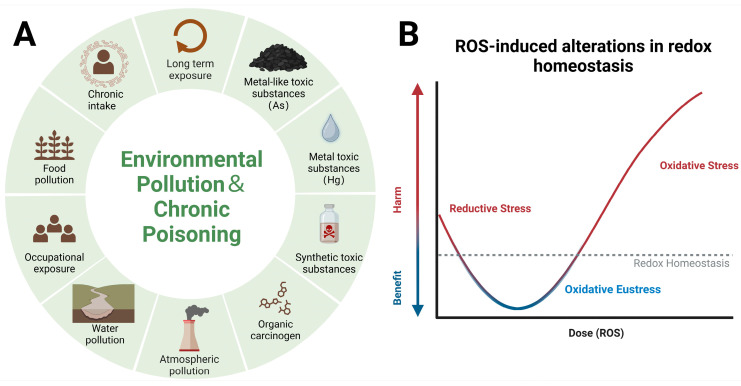
Carcinogenic factors in environmental pollution and chronic poisoning and changes in redox homeostasis during increased ROS levels. (**A**) Carcinogenic factors in environmental pollution and chronic poisoning: Under long-term exposure and chronic intake, metalloids (e.g., As), toxic metals (e.g., Hg), synthetic toxic substances (e.g., pesticides), and organic toxins (e.g., formaldehyde) can exhibit strong carcinogenic potential. Additionally, the sources of these toxic substances may include water, food, or air, as well as factors related to occupational exposure. This is also associated with long-term groundwater and crop contamination. (**B**) Changes in redox homeostasis during increased ROS levels: Under normal physiological conditions, the oxidative and reductive pressures in the body should be in a dynamic balance, with oxidants and antioxidants relatively balanced. In the case of pathological ROS increase, the reductive capacity will be progressively limited by the oxidants. When the oxidative stress exceeds the body’s maximum reductive capacity, it will induce related pathological outcomes. Created with BioRender.com (accessed on 22 July 2024). As: arsenic; Hg: mercury.

Oxidative stress (OS) primarily mediates oxidative damage, which occurs when the formation of ROS exceeds the antioxidant defense capacity of a cell [9,11]. While physiological concentrations of ROS play key roles in intracellular and intercellular signaling, cell growth and differentiation, excessive ROS induced by pathological processes such as injury and inflammation can lead to an imbalance in oxidative stress homeostasis and trigger a series of chain reactions [12,13,14]. Metabolic disorders and mitochondrial damage are common consequences of oxidative stress homeostasis imbalance. Regardless of the cause, mitochondrial damage reduces ATP production, leading to bioenergetic failure and potential mitochondrial toxicity [15,16,17]. Insufficient energy affects the function of sodium–potassium pumps, thereby altering the regular metabolic pathways within a cell [9]. Furthermore, a reduction in ATP production from the electron transport chain increases the reliance on glycolysis, resulting in the inhibition of the tricarboxylic acid cycle, accumulation of lactate, and decrease in intracellular pH. This state facilitates the transmission of nociceptive signals by activating receptors and channels such as TRPV1, ASIC, and P2X [18] (Figure 2 and Figure 3B).

Prolonged exposure to excessive ROS leads to mitochondrial dysfunction, endoplasmic reticulum stress (ER stress), the unfolded protein response (UPR), impaired proteasome function, and dysregulation of autophagy regulated by peroxisomes, causing a series of uncontrolled reactions [19]. Excessive ROS also increase vascular permeability, promote leukocyte adhesion, and lead to long-term changes in endothelial signaling and redox-sensitive transcription factors [20]. Persistent stimulation, altered metabolic pathways, and heightened glycolysis are high-risk factors for carcinogenesis.

In this context, the application of antioxidants appears to be an effective means of preventing carcinogenesis. Indeed, when pathological reactions occur, in addition to the natural antioxidant barriers within the body, the application of exogenous antioxidants becomes particularly important [21,22]. As an easily accessible and powerful antioxidant, ALA is widely chosen for its superior antioxidant properties.

- ALA and DHLA exhibit a high redox potential (320 mV), which enables more effective maintenance of the reductive state within the body compared with that observed for the endogenous reduced/oxidized glutathione (GSH/GSSG) system (240 mV). Additionally, these molecules interfere with various signaling pathways activated by oxidative stress, preventing the excessive production of reactive oxygen species [4,23];
- ALA can directly quench various reactive species, including ROS, reactive nitrogen species, hydroxyl radicals (HO^•^), hypochlorous acid (HclO), and singlet oxygen (^1^O_2_); it provides indirect antioxidant protection through metal chelation (ALA primarily binds Cu^2+^ and Zn^2+^, while DHLA can bind Cu^2+^, Zn^2+^, Pb^2+^, Hg^2+^, and Fe^3+^) and the regeneration of certain endogenous antioxidants, such as vitamin E, vitamin C, and glutathione [4,5,23];
- Many antioxidants exhibit limited bioavailability due to their instability in the bloodstream or hydrophilicity, which restricts their passage through cell membranes. However, ALA and DHLA exhibit amphipathic properties, allowing them to be distributed in both hydrophilic environments (e.g., plasma) and lipophilic environments (e.g., cell membranes). This enables ALA to function extracellularly and to protect key intracellular molecules [4] (Figure 2).

### 2.2. Anti-Infective and Anti-Carcinogenic Effects of Chronic Inflammation

Infections are considered one of the primary causes of cancer worldwide, and the prevention and treatment of infectious diseases have a significant impact on cancer incidence [25]. As indicated in the World Cancer Report 2018, 13% of cancer diagnoses can be attributed to infections [26]. While the carcinogenic process of pathogen infection can be complex, it can be simplified as follows: certain pathogens have direct effects on genes (mutations) and chromosomal rearrangement. For instance, the E7 protein of the human papillomavirus (HPV) binds to the retinoblastoma tumour suppressor (Rb) and cyclin-dependent kinase inhibitor p21, thereby promoting DNA replication and cell proliferation [27]. Similarly, hepatitis B virus (HBV) induces the activation of hypoxia-inducible transcription factors (HIFs), thus resulting in indirect carcinogenic effects [28]. For most pathogens, indirect carcinogenic effects are predominant, including the following:

- Chronic inflammation induced by infection can activate signaling pathways such as the NF-κB, JAK/STAT, and Rb pathways, promoting the synthesis of inflammatory factors and cellular stress states, leading to somatic mutations and/or oncogene activation;
- Pathogen-secreted byproducts and/or metabolites may alter local environmental conditions (e.g., redox homeostasis, pH levels) to favor their replication, often resulting in chromosomal DNA damage in the affected tissues or organs;
- Some pathogens possess the capacity to modify the immune microenvironment, resulting in the induction of disordered immune responses. This phenomenon has been observed in the co-induction of malignant B-cell clones by malaria parasites and Epstein-Barr virus (EBV), which produce immunosuppressive cytokines that induce T-cell apoptosis and/or trigger the recruitment of myeloid-derived suppressor cells and Tregs, as observed in the cytokine storm induced by COVID-19;
- Persistent infection symptoms can maintain local tissues in a state of constant stimulation and healing after damage, which may potentially lead to genetic mutations, epigenetic changes, and protein modifications. These changes may result in the activation of oncogenes and the inhibition of anticancer processes. Furthermore, prolonged inflammatory responses may also result in immune system exhaustion, thereby facilitating the escape of tumour cells from immune surveillance [29,30,31,32].

Furthermore, ALA can exert multiple beneficial effects in the following contexts:

- As an antagonist of various inflammatory pathways, ALA can inhibit the inflammatory activities mediated by NF-κB, TNF-α, and IL-6 and enhance the activity of the anti-inflammatory protein nuclear factor erythroid 2–related factor 2 (Nrf2), thereby reducing tissue damage [33,34,35];
- ALA helps maintain redox homeostasis, preventing the redox state from being skewed in favor of pathogen proliferation [36];
- ALA can regenerate depleted glutathione (GSH) during viral replication and internalization. The endogenous antioxidant glutathione is associated with leukocyte proliferation, enhanced immune system function, and effective antiviral protection. A reduction in glutathione levels can impair the Na^+^/H^+^ antiporter, resulting in a reduction in intracellular pH and creating an environment conducive to viral replication [36,37];
- ALA can increase TCA cycle activity, enhancing ATP production and providing energy for immune activities and healing. Additionally, inflammation suppression can indirectly enhance immune system function, preventing hypersensitive reactions such as cytokine storms [38].

Notably, physiological concentrations of ROS are also part of the anti-infective defense within the body, possessing both antimicrobial activity and the ability to synergize with nitric oxide (NO) to kill internalized pathogens [39,40,41]. This suggests that exogenous antioxidants such as ALA may be most beneficial during acute or chronic infections, while prophylactic or high-dose use might not yield significant benefits.

### 2.3. Effects of Detoxification on Environmental Pollution and Chronic Toxicity

The toxic effects of xenobiotics are a significant and pressing issue in environmental science and medicine, particularly against the backdrop of various external pollutants [4,19,42,43]. Naturally occurring toxic metals and metalloids (e.g., arsenic, mercury, chromium, and lead), synthetic toxic substances (e.g., pesticides), and organic toxins (e.g., formaldehyde) all demonstrate high carcinogenic potential under conditions of long-term exposure and chronic toxicity [44,45,46]. This means that regardless of ingestion through water, food, or air or factors such as occupational exposure, a variety of toxic substances can increase the risk of cancer over prolonged periods [47,48,49]. Research indicates that the primary carcinogenic mechanisms of most toxins involve the production of excessive ROS, inhibition of antioxidant pathways (such as Nrf2/ARE), and/or interference with normal antioxidant functions (such as glutathione), leading to oxidative stress, inflammation, mitochondrial and DNA damage, and even cell death [50,51]. Moreover, certain toxins, such as arsenic, not only target mitochondria as a primary site of toxic action but also increase carcinogenic risk by altering epigenetic modifications or causing critical DNA hypermethylation through the p53 protein [52,53,54,55,56,57] (Figure 3A).

As a detoxifying agent, ALA is effective at counteracting various toxins. The detoxifying actions of ALA can be attributed to its antagonistic interactions with multiple toxins:

- ALA can scavenge the excessive ROS generated by poisoning, maintain redox balance, and reduce damage to crucial structures such as DNA and mitochondria;
- For metal toxins, ALA has chelating properties, reducing the cellular uptake of toxic metals and potentially promoting their excretion by forming complexes [58];
- ALA facilitates the regeneration of reduced glutathione through the Nrf2/ARE signalling pathway, thereby reducing the interference with normal antioxidant functions caused by toxins;
- ALA has been demonstrated to be particularly effective in the detoxification of mercury. In addition to chelating Hg^2+^, ALA can mitigate mercury toxicity by interacting with selenide compounds. This interaction results in the formation of stable complexes with thiol groups in selenium enzymes such as thioredoxin reductase, leading to mercury separation [59,60];
- ALA can also work synergistically with dimercaptosuccinic acid (DMSA), a known heavy metal detoxifier, to lower arsenic levels in rat models [61].

Based on current research data, ALA appears to be a promising detoxifying agent and may play a role in cancer prevention. Interestingly, the harmfulness of the atmospheric pollutant PM2.5 has been shown to be due not only to its concentration but also to the toxic substances it contains [62,63]. Developing countries such as South Africa, China, and Bangladesh have reported long-term challenges with heavy metal pollution in groundwater and crops, highlighting the urgent need for widespread application of detoxifying agents [64,65]. However, importantly, there is some conflicting evidence regarding the ability of ALA to reduce mercury levels in the kidneys or brains of animal models. Furthermore, there are currently no human detoxification trials involving ALA/DHLA, and the conclusions drawn are primarily based on in vitro experiments; therefore, further data are needed for validation. Additionally, the protective effect of preventive ALA administration against cancer has not yet been robustly validated in clinical trials [66].

### 2.4. Anti-Carcinogenic Effects against Other Carcinogenic Factors

ALA also has potential indirect anti-carcinogenic effects. For example, it can help stimulate insulin production and increase tissue sensitivity to and utilization of glucose, thereby reducing insulin resistance and improving insulin sensitivity and blood sugar control [67,68]. Clinical trials have demonstrated that diabetes is a risk factor for the development of cancer. Individuals with fasting blood sugar levels greater than 7.8 mmol/L have a 25% greater risk of dying from cancer, mainly those with liver cancer, pancreatic cancer, and breast cancer, than those with fasting blood sugar levels less than 5.6 mmol/L [69]. By improving insulin sensitivity and blood sugar control, ALA may reduce the risk of the development of these cancers to some extent.

Additionally, the anti-metabolic disorder effects of ALA are also evident in the correction of carcinogenic factors resulting from conditions such as obesity. A meta-analysis of the impact of ALA on weight and BMI demonstrated that the mean weight and BMI reductions observed in the ALA group were significantly greater than those observed in the placebo group [70]. This weight loss effect can help achieve the overall goal of combating cancer, particularly in conditions such as non-alcoholic fatty liver disease; furthermore, it aids in alleviating social and personal issues resulting from psychological pressure or discrimination due to obesity.

## 3. ALA and Its Anticancer Effects

**Figure 4:**
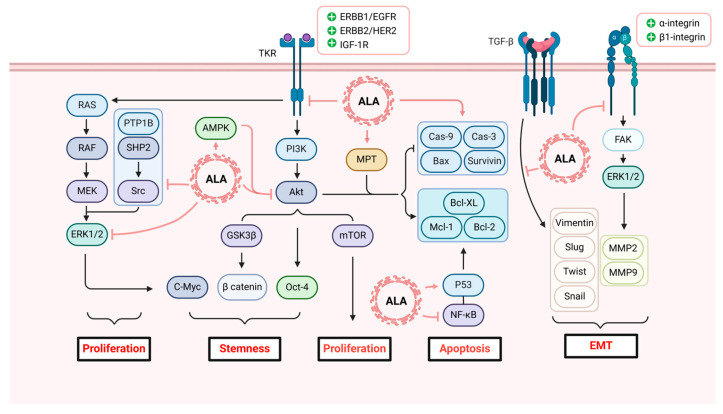
Effects of ALA on several signaling pathways implicated in tumorigenesis. Proliferation and growth: Cell proliferation and growth are dominated by TKRs, where the overexpression of ERBB1/EGFR, ERBB2/HER2, and IGF-1R activates carcinogenic signaling pathways such as PI3K/Akt/mTOR and MAPK/ERK. ALA targets ERBB1/EGFR to exert anticancer effects primarily by inhibiting the generation and release of downstream effectors. ALA can also target and inhibit PTP1B and SHP2 to control Src kinase, thereby affecting the ERK pathway. Additionally, ALA targets IGF-1R, limiting its maturation. Furthermore, ALA directly limits the phosphorylation of ERK and Akt. Tumor stemness and cancer stem cells: The activation of the Akt pathway has been demonstrated to significantly induce stemness, resulting in the phosphorylation and inactivation of GSK3β. This, in turn, leads to the stabilization of β-catenin and the phosphorylation activation of Oct-4. ALA inhibits the activation of tumour stem cells by reducing Akt phosphorylation. Furthermore, the inhibition of Akt phosphorylation affects the activity of C-Myc, thereby exerting additional control over tumour stemness. Apoptosis: The process of apoptosis is regulated by the coordinated action of pro-apoptotic and anti-apoptotic proteins. ALA can induce the inhibition of anti-apoptotic proteins (such as Mcl-1, bcl-Xl, bcl-2, Survivin) and the expression of pro-apoptotic proteins (such as Cas-9, Cas-3, Bax). ALA also stimulates the mutant p53 protein and depletes MGMT by inhibiting NF-κB signalling, thereby inducing apoptosis. Furthermore, ALA induces MPT, which also results in apoptosis. Metastasis and EMT: The EMT process, which is responsible for cell migration and invasion, is regulated by TGF-β and FAK. ALA inhibits FAK activation by downregulating β1-integrin expression and reduces the levels of MMP-9 and MMP-2. In addition, ALA inhibits the expression of EMT markers, including Snail, vimentin, and Zeb1 [71]. (Arrows indicate promotion, lines indicate inhibition). Created with BioRender.com (accessed on 22 July 2024). TKRs: tyrosine kinase receptors; IGF-1R: insulin-like growth factor 1 receptor; ERBB1/EGFR: epidermal growth factor receptor; ERBB2/HER2: human epidermal growth factor receptor 2; ERK: extracellular signal-regulated kinase; AMPK: AMP-activated protein kinase; PI3K: phosphoinositide 3-kinase; Akt: protein kinase B; GSK3β: glycogen synthase kinase 3 beta; C-Myc: cellular myelocytomatosis; Oct-4: octamer-binding transcription factor 4; mTOR: mammalian target of rapamycin; MPT: mitochondrial permeability transition pore; Bax: Bcl-2-associated x protein; Cas-9: caspase-9; Cas-3: caspase-3; Bcl-2: B-cell lymphoma 2; Mcl-1: myeloid cell leukemia 1; Bcl-XL: B-cell lymphoma-extra large; MGMT: O6-methylguanine-DNA methyltransferase; NF-κB: nuclear factor kappa-B; FAK: focal adhesion kinase; MMP: matrix metalloproteinase; TGF-β: transforming growth factor beta.

ALA can exert direct anticancer effects on the processes of proliferation, cycle, apoptosis, and metastasis involved in cancer development. Figure 4 summarizes the pathways of ALA’s effects on cancer. 

### 3.1. Anticancer Cell Proliferation

The proliferation of tumor cells is primarily driven by TKRs, including members of the ERBB family (such as ERBB1/EGFR, ERBB2/HER2) and insulin-like IGF-1R, which activate oncogenic signalling pathways, including the PI3K/Akt/mTOR and MAPK/ERK pathways when overexpressed [71]. Based on this scenario, various studies have shown that ALA can target ERBB1/EGFR to exert anticancer effects in cancer types such as thyroid cancer and breast cancer, primarily by inhibiting the generation and release of downstream effectors [72,73]. Furthermore, ALA has been demonstrated to inhibit the activity of the PTP1B and SHP2 enzymes [74]. The protein tyrosine phosphatases PTP1B and SHP2 have been demonstrated to promote the Src kinase and Ras/Erk pathways, and together constitute a significant proportion of HER2/Neu signalling [75]. Based on this mechanism, one of the anticancer mechanisms of ALA involves reducing tumor cell activity at the TKR level and inhibiting tumor cell proliferation mediated by the oncogenes ERBB1/EGFR and ERBB2/HER2.

Multiple studies have shown that the regulation of ROS by ALA can also influence cancer cell proliferation. For example, breast cancer cells exposed to ALA accumulate immature insulin-like growth factor 1 receptor (pro-IGF-1R) in the cytoplasm, indicating that IGF-1R is not properly positioned on the cell membrane and is thus inactive [76]. In further studies, ALA was found to reduce the activity of furin, a precursor protein that promotes IGF-1R maturation, thereby affecting the subsequent maturation process of IGF-1R [76]. The cAMP response element-binding protein (CREB) is a transcription factor that induces furin expression, and its activity is primarily limited by ROS production [10,77]. ALA, on the other hand, has been found to increase ROS production, thereby inducing CREB/furin inhibition and preventing IGF-1R internalization and maturation. As an upstream regulatory factor, immature IGF-1R loses its ability to activate the PI3K/Akt/mTOR and MAPK/ERK pathways, thereby limiting tumor cell proliferation [78,79]. Surprisingly, the inhibitory effect of ALA also involves another TKR that is highly homologous to IGF-1R, namely, the insulin receptor (IR). This synergistic effect induces the simultaneous dysfunction of immature IGF-1R and IR, thereby enhancing tumor suppression [10].

In addition to TKRs, ALA has been found to directly target downstream pathways. A recent in vitro study on breast cancer revealed that ALA can directly target the PI3K/Akt/mTOR and MAPK/ERK proliferation pathways, restricting the phosphorylation of ERK and Akt [76]. Moreover, ALA can activate antagonistic AMPK to further enhance the inhibitory effect on Akt and suppress mTOR [80,81,82,83]. Notably, CSCs, due to their self-renewal capacity and differentiation potential, are regarded as the primary instigators of malignant tumour occurrence, metastasis, and drug resistance. Akt phosphorylation represents a crucial step in maintaining the stemness of tumor cells and thus represents a promising target for therapeutic intervention [78]. The inhibition of Akt phosphorylation results in the suppression of the phosphorylation and activation of β-catenin and Oct-4. This, in turn, affects the activity of stem cell populations and limits the proliferation, differentiation, and metastasis of cancer cells [10,78]. The inhibitory effect of ALA on CSCs is advantageous, as although many advances have been made in cancer treatment methods, CSCs may remain unaffected in those approaches. This inhibitory effect has been validated in an experiment on non-small cell lung cancer [84,85]. Furthermore, phosphorylated ERK regulates C-Myc, which coordinates a multitude of biological processes in tumor stem cells, including cell metabolism, self-renewal, differentiation, and growth [86,87].

ALA can also promote the expression of the p53 protein. p53 is a tumor suppressor that plays a crucial role in regulating cell cycle checkpoints, DNA repair, and apoptosis. Elevated levels of p53 can arrest the cell cycle at the G1 phase, thereby constraining the proliferative capacity of tumor cells and inducing apoptosis, among other adverse outcomes [88].

### 3.2. Pro-Apoptotic Effects on Cancer Cells

Before discussing the pro-apoptotic effects of ALA on tumor cells, it is essential to clarify that the roles and mechanisms of ROS in inducing cell damage and apoptosis differ between cancerous and normal cells [88]. In normal tissues, the presence of elevated levels of ROS may facilitate the transformation of normal cells into cancer cells, thereby contributing to the development of cancer. However, due to their excessive and frequent proliferative activities, cancer cells typically exhibit high levels of ROS [89]. To survive and function in this highly oxidative environment, cancer cells manage ROS levels through effective antioxidant mechanisms to prevent ROS accumulation beyond the apoptotic threshold [90,91,92]. Therefore, inducing ROS production to trigger apoptosis appears to be more advantageous than reducing ROS levels within the cancer cell microenvironment. This is the fundamental mechanism of chemotherapy.

Interestingly, research has shown that the direct anticancer effect of the antioxidant ALA is manifested as an increase in intracellular ROS levels in cancer cells [10]. This pro-oxidant characteristic may stem from the distinct cellular environments of normal and cancer cells. By increasing ROS levels, ALA effectively exploits the differences in oxidative stress response mechanisms and sensitivities between cancer and normal cells, altering the balance of anti-apoptotic and pro-apoptotic proteins to selectively induce apoptosis in cancer cells [39]. For instance, in hepatocellular carcinoma, ALA has been found to trigger the intrinsic apoptotic pathway by activating caspase-9 and caspase-3, inducing endoplasmic reticulum (ER) stress and the UPR and further enhancing apoptosis [93,94,95]. In breast cancer and leukemia, ALA dose-dependently activates caspase-3 and upregulates the expression of the pro-apoptotic protein Bax while downregulating the expression of anti-apoptotic proteins such as Mcl-1, Bcl-2, and Bcl-XL. These effects have also been confirmed in several studies on ovarian cancer [95]. Additionally, in colon cancer, ALA has been found to stimulate mutant p53 protein and deplete MGMT protein by inhibiting NF-κB signaling [96,97]. Mutant p53 is known as an apoptosis activator that induces the transcription of pro-apoptotic genes, thus triggering intrinsic apoptosis. Additionally, MGMT is an essential molecule for DNA repair and acts as a barrier protecting cells from autophagy induced by alkylating agents [98,99,100].

Moreover, ALA can activate the mitochondrial permeability transition pore (mPTP), which is typically inhibited in cancer environments, thereby inducing MPT [101]. This process alters mitochondrial permeability, restricts mitochondrial function, reduces ATP production, and induces apoptosis. Paradoxically, ATP is a critical component of apoptosis, and that the loss of mitochondrial ATP synthesis due to MPT might not permit MPT-driven apoptosis. In reality, MPT may not affect the entire mitochondrial network within a cell but could occur at the level of individual mitochondria, indicating that this process might proceed in a multistage manner from local to global, ultimately leading to cell apoptosis. Thus, even with a reduction in ATP, MPT-induced apoptotic events can continue [102,103,104].

### 3.3. Anti-Migration, Invasion, and EMT Effects

Tumor metastasis is a complex, multistep process in cell biology known as the invasion-metastasis cascade. This process involves cancer cells invading surrounding extracellular barriers and basement membranes, infiltrating into vascular lumens, extravasating into distant organs, colonizing, and adapting to foreign tissue microenvironments [105,106,107]. This entire process is regulated by two important pathways: the TGF-β and FAK pathways [71,103,104,108]. These pathways also induce a critical step for metastasis, namely, EMT. The EMT process imparts an epithelial phenotype to cancer cells, enhancing their invasive and migratory capabilities. EMT-related transcription factors, such as vimentin, Slug, Twist, and Snail, are further induced to reinforce this process, while the expression of cell adhesion molecules such as E-cadherin, which act as barriers to metastasis, is downregulated [73,85,102,104,109].

Based on current knowledge, ALA can inhibit the TGF-β signaling pathway to curb the progression of EMT, thereby affecting tumor metastasis [110]. In a study on breast cancer, ALA-treated MDA-MB-231 and 4T1 breast cancer cell lines both exhibited downregulated expression of EMT markers such as Snail, vimentin, and Zeb1, along with impaired cell migration capabilities [73]. A similar outcome was observed in another study focusing on ovarian cancer [111]. In addition to TGF-β, ALA was also found to limit the adhesion step of tumor metastasis mediated by FAK by downregulating β1-integrin expression [112]. In studies on prostate cancer, ALA was shown to inhibit invasion by decreasing the mRNA levels of key matrix metalloproteinases (MMPs), specifically MMP2 and MMP9, which are crucial for the metastatic process [113].

Moreover, as mentioned above, ALA has the potential to limit the abilities of cancer stem cells, which are closely associated with EMT [109,114,115]. EMT is not only involved in the differentiation of pluripotent stem cells into the three embryonic germ layers during early development but also plays a role in the acquisition of adaptive traits by cancer cells during the malignant process [116,117,118]. This suggests that the inhibitory effects of ALA can offer multiple benefits. In addition to restricting EMT-mediated metastasis, ALA also reduces the differentiation and drug resistance mediated by cancer stem cells [119].

### 3.4. Unproven Hypotheses and Other Effects

In the context of cancer, the majority of solid tumours exhibit hypoxic characteristics due to the rapid growth of these tumours, which outpaces the oxygen supply. This condition is further exacerbated by irregular tumor blood vessel formation and poor circulatory dynamics [120,121,122]. Under hypoxic conditions, HIFs are activated to assist cancer cells in surviving in low-oxygen environments [123]. Among these, HIF-1α primarily regulates anaerobic glycolysis and cell survival, while HIF-2α regulates erythropoietin (EPO) and tumor stemness or pluripotency [124]. Current evidence confirms that homozygous mutations in ALA synthase (LIAS), a key precursor in ALA synthesis, activate HIF expression, suggesting that endogenous ALA may alleviate the hypoxic environment within tumors [125,126]. Furthermore, the exogenous application of ALA has been demonstrated to limit the expression mechanism of the PI3K/Akt/mTOR pathway, which acts as an upstream regulatory pathway of HIF-1α. This effect is consistent with that observed for apigenin and genistein, which have been identified as specific inhibitors of HIF-1α [127]. Therefore, it is inferred that lipoic acid may inhibit the expression of HIF-1α and act as a synergistic agent with natural polyphenolic substances such as apigenin and genistein, thereby enhancing their inhibitory effects on cancer. Its mechanism of action against cancer might involve creating a more hypoxic environment and reducing the ability of cancer cells to tolerate hypoxic conditions, thereby inducing their death and simultaneously reducing issues such as metastasis and drug resistance associated with hypoxia. However, definitive evidence to support this hypothesis is still needed.

In addition to HIF-1α, studies on genes upstream of ALA and other chronic diseases suggest that metal chelation is also one of the primary mechanisms of action of ALA. Metal ions, such as copper and iron, as part of electron transfer and cellular respiration, accumulate at different levels in normal and cancer cells [128,129]. Cancer cells are known to have increased iron levels due to their frequent and active cellular activities. Tumor cells control iron content by secreting iron-containing exosomes and other means to avoid reaching levels that trigger ferroptosis [130]. Copper is mainly associated with HIF-α and the promotion of tumor angiogenesis, creating a positive feedback loop [131]. However, copper accumulation can also trigger mitochondrial-associated cell death, known as cuproptosis [128]. Based on this background, it can be hypothesized that ALA might facilitate the accumulate iron and/or copper through metal chelation or pro-oxidative effects to reach thresholds that induce ferroptosis or cuproptosis, thereby inducing cancer cell death. Although this hypothesis seems theoretically plausible, clear evidence is needed for validation.

Combining these two hypotheses, it can be hypothesized that ALA may regulate copper and HIF-2α to limit tumor angiogenesis. The expression of genes related to EPO and tumour stemness or pluripotency is primarily regulated by HIF-2α, and copper is also involved in angiogenesis. In diabetic experimental models, ALA has been shown to have antagonistic effects on VEGF, EPO, and angiopoietin-2, which are proteins related to angiogenesis [132]. The significance of VEGF, EPO, and angiopoietin-2 in tumor nutrition and metastasis is well documented, suggesting that ALA may exert a limiting effect on tumor growth and metastasis. However, these hypotheses require further research and validation.

## 4. The Adjuvant Role of ALA in Cancer Treatment

### 4.1. Enhancing Chemotherapy and Radiotherapy Effects

Tumors have the potential to develop resistance to current drugs or treatments, which becomes increasingly evident as treatment progresses. Although radiotherapy is a primary treatment modality for both early-stage and late-stage breast cancer, its application is often limited by radiation resistance, recurrence, and metastasis [133]. This limitation is primarily due to EMT activation in tumor cells following radiation damage, subsequently increasing the migration, invasion, and metastasis propensity of cancer cells [134,135]. Studies have shown that the pretreatment of breast cancer cells with ALA can inhibit the EMT phenomenon induced by radiotherapy, mainly by restricting the expression of TGF-β1 [136,137]. Furthermore, ALA has been shown to enhance the efficacy of the chemotherapeutic drug paclitaxel in breast and lung cancer cells by inhibiting the NF-κB signalling pathway and the functions of integrin β1/β3 [138,139]. This mechanism suggests that ALA pretreatment may serve as an effective method to prevent the emergence of drug-resistant tumors. Moreover, research has demonstrated that ALA can enhance the functionality of various other anticancer drugs, including 5-fluorouracil in colon cancer cells and cisplatin in MCF-7 breast cancer cells [140].

Notably, the overall trend of ALA activity remains unclear, especially considering the influence of the dynamic tumor microenvironment (e.g., its antioxidative effects in normal cells versus its pro-oxidative effects in cancer cells). The efficacy of exogenous antioxidants in safeguarding tissues from oxidative stress within the body is contingent upon a multitude of variables, including the antioxidant type, antioxidant pharmacokinetic properties, antioxidant concentration at the site of action, and the nature of oxidative stress. Therefore, it is speculated that the anticancer effects of ALA may be related to cancer stage. Changes in the microenvironment seem to provide opportunities for the effects of ALA to be altered when other anticancer treatments (such as chemotherapy, radiotherapy, and surgical resection) are applied to treat or after anticancer treatments have been administered to treat a tumor. Further research is necessary to substantiate this hypothesis.

### 4.2. Reducing Adverse Effects after Anticancer Treatment

With advancements in cancer treatment and the prolonged survival of patients, the attention given to adverse reactions posttreatment has intensified, posing challenges to clinical cancer therapy. Chemotherapy is a well-established method for extending the lives of cancer patients. Nevertheless, chemotherapy-induced peripheral neuropathy (CIPN) represents one of the most prevalent long-term toxic reactions [141]. Statistical data indicates that up to 80% of cancer patients undergoing cytotoxic chemotherapy experience varying degrees of CIPN. This condition is primarily characterized by persistent tingling, burning sensations, and numbness in the absence of harmful stimuli, often presenting in a “stocking-glove” distribution pattern [142,143,144]. CIPN significantly impacts the quality of life of patients and can sometimes lead to delays or interruptions in cancer treatment [145]. Therefore, there is a pressing necessity to minimise the incidence of adverse reactions such as CIPN while maintaining the efficacy of chemotherapy, thus enhancing the quality of life of patients.

A substantial body of evidence indicates that oxidative damage plays a role in the pathogenesis of CIPN. This is because chemotherapy drugs such as bortezomib generate high concentrations of ROS, which is a key factor in the induction of mitochondrial dysfunction, microtubule damage, demyelination, and cell apoptosis, leading to neuronal damage [16]. Furthermore, certain functional and structural attributes of the peripheral nervous system (PNS) increase susceptibility to the accumulation of chemotherapeutic drugs and certain neurotoxins. Mammals are more susceptible to oxidative stress due to their high phospholipid content, abundant mitochondrial axoplasm, and weaker cellular antioxidant defence capabilities [146,147].

Two in vitro experiments based on nab-paclitaxel (nab-PTX) have demonstrated the neuroprotective effects of ALA in models of CIPN. ALA has been demonstrated to enhance the expression of the essential mitochondrial protein frataxin, which has antioxidant properties, and the activation of the Nrf2 signalling pathway. This approach results in the rescue of chemotherapy-induced CIPN without promoting tumour growth or reducing the efficacy of the chemotherapy [148]. This effect has been validated in an in vivo study involving 14 cancer patients (10 with grade 2 CIPN and 4 with grade 3 CIPN) treated with docetaxel [149]. The oral administration of ALA significantly reduced the severity of CIPN in patients after a period of intravenous injection. However, another study evaluating the role of oral ALA in preventing oxaliplatin- or cisplatin-induced neurotoxicity yielded different results. Among the 243 patients who participated in the study, 70 (29%) completed the 24-week course of treatment. There was no significant difference in the incidence of CIPN pain between the ALA and placebo groups [150].

Notably, the credibility of several in vivo studies has been compromised due to high attrition rates resulting from poor dosing regimens. Oral ALA has a bioavailability of approximately 30% due to issues such as poor stability in the stomach, low solubility, and hepatic degradation. In contrast, the intravenous administration of ALA yields higher bioavailability [151]. This indicates the need for further research to develop a unified and efficient method of ALA intake to validate its efficacy as a treatment for CIPN.

### 4.3. Auxiliary Role in Cancer Nanomedicine

Due to inherent deficiencies in traditional cancer treatment methods, nanotechnology has been employed in cancer therapy to enhance efficacy, precision, and safety [152]. ALA, as a coupling agent, has been widely adopted due to its distinctive advantages:

- ALA is a member of the liposome family and possesses natural disulfide bonds that enable it to remain relatively stable in systemic circulation and rapidly degrade upon absorption by cancer cells, exerting its effects. Furthermore, ALA exhibits strong amphiphilicity, excellent biocompatibility, and easy chemical modification, rendering it one of the most promising candidates for anticancer drug delivery [153,154,155,156,157];
- In normal cells, ALA acts as an antioxidant by clearing ROS. However, in cancer cells, it can exert pro-oxidative effects, inducing pathways that restrict cancer progression. This indicates that nanostructures containing ALA, in conjunction with anticancer drugs, exhibit synergistic effects, increasing selectivity and providing protective and reparative functions against the side effects of several anticancer drugs that mediate ROS generation;
- ALA is a naturally occurring compound with nontoxic properties that effectively avoids the toxicity associated with drug accumulation caused by the enhanced permeability and retention (EPR) effect when it is administered systemically to treat solid tumors [158].

**Figure 5:**
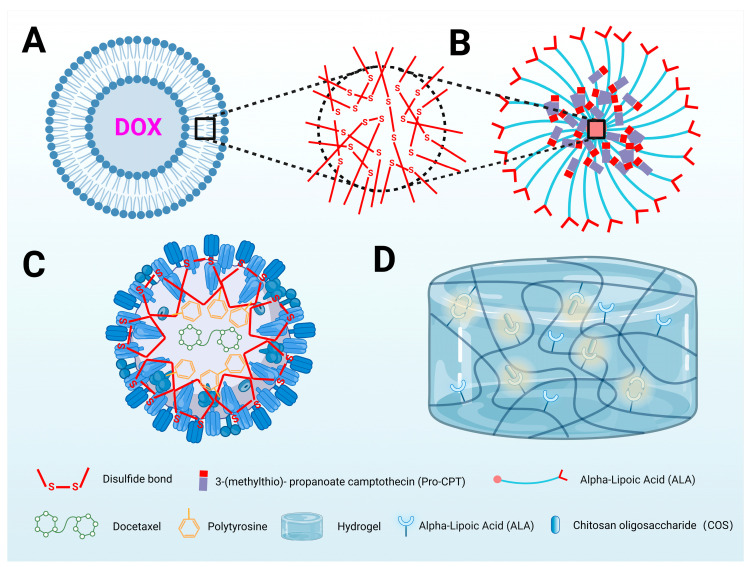
The pivotal role of ALA in the development of novel nanomedicine anticancer drugs. (A) Cross-linked di-LA-PC liposomes: ALA enhances the nanomedicine’s transport to target tissues through its amphiphilic nature and excellent biocompatibility with disulfide bonds. (**B**) Pro-CPT@cLANs: In this nanomedicine, ALA stabilizes the drug via disulfide bonds, which are cleaved by overexpressed GSH in tumor cells, effectively releasing Pro-CPT. ALA also serves to mitigate issues of toxicity associated with carriers. (**C**) DTX-CMHN: ALA forms the outer shell of DTX-CMHN, ensuring stability, amphiphilicity, and compatibility. This nanomedicine combines natural pTyr through π-π stacking interactions with doxorubicin and paclitaxel, achieving high drug loading and accelerated release within tumor cells. (**D**) COS@LA-hydrogel: The drug-loaded hydrogel incorporates chitosan COS into an ALA hydrogel, leveraging the unique location of skin cancer. ALA and COS synergize for anti-inflammatory, antioxidant, and anti-infective effects, addressing post-surgical residual cancer cells and inflammation to prevent cancer recurrence. ALA and COS are natural components with high biocompatibility [158,159,160,161]. Created with BioRender.com (accessed on 22 July 2024). DOX: doxorubicin; Cross-linked di-LA-PC liposomes: dimeric lipoic acid-derived glycerophosphorylcholine liposomes; Pro-CPT: 3-(methylthio)-propanoate camptothecin; ProCPT@cLANs: Pro-CPT loaded with cross-linked (R)-(+)-lipoic acid nanoparticles; GSH: glutathione; DTX-CMHN: cross-linked multifunctional hyaluronic acid nanoparticles carrying docetaxel; pTyr: phosphotyrosine; COS@LA-hydrogel: incorporating chitosan oligosaccharides into lipoic acid hydrogel; COS: chitosan oligosaccharides.

Several experiments have validated the anticancer auxiliary advantages of LA cross-linked nanoformulations. For instance, in an experiment targeting breast cancer cell lines, an ALA–glycerophosphocholine conjugate not only enhanced the efficiency of the loaded drug doxorubicin but also demonstrated potential to evade tumor resistance [158] (Figure 5A). In another study, nanoparticles loaded with paclitaxel exhibited a prolonged half-life, high efficiency, and antimetastatic properties [159] (Figure 5C). In in vivo experiments conducted with colon cancer-bearing mice, LA-crosslinked 3-mercaptopropionic acid camptothecin nanoparticles (Pro-CPT) retained compatibility, promoted anticancer activity, with synergistic effects, thus reducing the required drug dosage to achieve the same effect, and were safer [160] (Figure 5B). Notably, ALA itself can also act as a loaded anticancer drug and be delivered to target tissue via carriers, helping to increase the concentration in target tissues and aiding in altering the oxidative stress status of the microenvironment.

Intriguingly, two studies not directly targeting tumors have explored the anti-inflammatory and anti-postoperative recurrence effects of ALA. The studies employed strategies involving the sustained release of aspirin by ALA nanoparticles and the sustained release of chitosan oligosaccharide (COS) by ALA hydrogels to address the issue of recurrence caused by residual inflammation after chemotherapy or surgery [161,162]. Of particular interest is the application of the hydrogel anti-recurrence scheme following the surgical excision of skin cancer. The research primarily focused on the mutual promotion of residual cancer cells and inflammatory reactions after skin cancer excision, which induces tumor recurrence; notably, the specific location of skin cancer allows the drug-loaded hydrogel to have multiple effects, such as protection, anti-inflammatory properties, the promotion of healing, and a reduction in infection (Figure 5D).

Despite the numerous advantages of nanomedicine, there is still a considerable distance to traverse before its application in the human body can be considered fully realized. Compared with traditional therapies, existing liposomal therapeutic agents have not improved overall survival (OS) rates. Furthermore, phase III clinical trials evaluating liposomal encapsulated cytarabine-daunorubicin (Vyxeos), which is believed to improve OS in patients with acute myeloid leukemia, have just completed [163]. Another commercially available nanodrug is albumin-bound paclitaxel (Abraxane), which is superior to paclitaxel in terms of the tumor response rate and response time in breast cancer patients when administered every three weeks. However, it did not show the same advantages in terms of nondisseminated tumors or overall survival when administered weekly, and it increased toxicity. Two additional types of nanotherapeutic agents, polymer vesicles (such as NK105) and polymer nanoparticles (such as CRLX101 and BIND-014), have yielded disappointing clinical trial results [164,165,166]. Consequently, the development strategy for nanomedicines nanomedicine may need to be re-evaluated, including more precise patient selection to identify those most likely to respond to such treatment.

### 4.4. Other Auxiliary Effects

Among the auxiliary effects classified as “other”, the primary objective is to enhance the quality of life of cancer patients.

Cancer-induced neuropathic pain (NP) is defined as a chronic change in somatosensory system function caused by injury or pathological disorders, which can cause suffering for many cancer patients, particularly those with pancreatic cancer [9]. Although the precise mechanisms underlying NP remain unknown, mounting evidence suggests a correlation between NP and oxidative stress, suggesting that the use of ALA may be a promising avenue for further investigation. ALA has been shown to inhibit NADPH oxidase, a key enzyme closely associated with NP, including NOX4 [167]. While research on the analgesic function of ALA is currently limited to aspects related to diabetic neuropathy, its potential as a means to alleviate cancer-induced NP reactions cannot be overlooked.

Furthermore, individuals diagnosed with cancer are more prone to inactivity when confronted with psychological distress, social isolation, and physical deterioration associated with the disease. This phenomenon results in a decline in exercise capacity and the emergence of a range of physical ailments. Studies have demonstrated that pretreatment with an antioxidant mixture (300 mg of ALA, 500 mg of vitamin C, and 200–400 IU of vitamin E) in patients with long-term exercise restrictions can enhance exercise-induced total leg blood flow. Another study on sedentary elderly individuals indicated that the preingestion of antioxidants (300 mg of ALA, 500 mg of vitamin C, and 200–400 IU of vitamin E) before handgrip exercise reduces the production of ROS and restores exercise-induced brachial artery vasodilation [168,169,170]. This suggests that the use of antioxidants may be a potential means of helping to restore exercise adaptability in situations of exercise limitation for pathological reasons.

Moreover, the role of antioxidants such as ALA in reperfusion injury in acute and chronic ischemia or transplant patients has also garnered increasing attention [31]. Reperfusion following ischemia induces significant Ca^2^⁺ influx and generates large amounts of oxygen free radicals [171]. Ischemia/reperfusion injury may lead to posttransplant organ rejection or failure in cancer patients through the stimulation of inflammatory pathways. The use of antioxidants, such as ALA, appears to mitigate or alleviate this issue to some extent, particularly in ROS-sensitive cells, such as neurons.

## 5. Future Prospects and Questions

Further research is required to elucidate the mechanisms of action of ALA in cancer and to assess its environmental effects and efficacy in depth.

In terms of clinical application, nanomedicine appears to be the most promising approach for leveraging the anticancer effects of ALA. Nanocarriers, which are specifically designed to possess characteristics such as sustained release, selectivity, and diversity, can effectively address issues inherent in the traditional application of ALA, such as poor bioavailability, effectiveness, and absorption rates, short half-life, and limited efficacy when used alone. However, due to the complex structure and intricate manufacturing processes involved, further research is needed to demonstrate the effectiveness of these carriers on a large scale. Previously, it was speculated that more efficient and sustained ALA suspensions or drug delivery materials, such as hydrogels, and the utilization of various forms of local administration (such as ALA washes, eye drops, etc.) may amplify the effects of ALA. Collaboration from multiple parties will still be necessary in the future to enhance the advantages of ALA-related nanomedicines.

Furthermore, as mentioned above, ALA can be divided into exogenous and endogenous forms. Endogenous ALA is synthesized through a cellular de novo pathway, which involves mitochondrial fatty acid synthesis (mtFAS), followed by a reaction catalyzed by an ALA-specific enzyme, ALA synthetase 2 (LIPT2), and a reaction catalyzed by a second enzyme, LIAS, which is the rate-limiting enzyme in the entire synthesis process. Finally, LIPT1 transfers the synthesized ALA to other targets [172]. Nevertheless, endogenously synthesized ALA cannot be replaced by exogenous ALA in certain physiological activities, such as mitochondrial coenzyme action. In light of this background, it is pertinent to inquire, through bioinformatics and subsequent basic experimental validation, we have learned that in numerous cancer types, including liver cancer, LIPT2, LIPT1, and LIAS, which act as precursors for ALA synthesis, exhibit oncogenic activity [173]. Conversely, exogenous supplementation of ALA has been shown to inhibit tumor cell proliferation. We hypothesize that this contradictory result may be due to differences in the effects of endogenous and exogenous ALA. In other words, endogenously synthesized small doses of ALA can act as antioxidants in cancer cells to prevent ROS from reaching the apoptosis threshold, chelating metals, and promoting angiogenesis. Alternatively, small doses of ALA may also exert pro-oxidant effects, but the concentration may not be sufficient to induce ROS accumulation and may only help cancer cells maintain a high oxidative environment.

Importantly, endogenous ALA participates in mitochondrial activity and acts as a crucial substrate for the tricarboxylic acid cycle, a function that exogenous ALA does not possess. According to the latest research findings, the tricarboxylic acid cycle also plays an important role in the progression of cancer [174]. Based on this theory, the endogenous ALA synthesis pathway may serve as a key condition for maintaining the tricarboxylic acid cycle in cancer cells.

Further research is required to address the aforementioned issues and to explore the mechanisms of action of endogenous and exogenous ALA, with the aim of elucidating their roles in cancer development.

## 6. Conclusions

In summary, ALA is a highly promising multifunctional anticancer molecule. It exhibits antitumor activity in several cancer models, reducing the influence of carcinogens; regulating multiple signaling pathways associated with cancer cell proliferation, apoptosis, invasion, migration, EMT, and cancer stemness; enhancing the efficacy of radiotherapy and chemotherapy; reducing the side effects of tumor treatment; and assisting other therapeutic drugs. Nevertheless, the precise mechanisms of action of ALA remain to be fully elucidated, and further research is necessary to enhance our understanding of its potential anticancer effects.

One of the current challenges is to clarify the functional differences between endogenous and exogenous ALA, while another key aim is to explore the mechanisms by which ALA exerts its multiple effects and the opportunities for action transformation. Additional studies may provide crucial data for the design of combination ALA therapy, with the aim of enhancing the efficacy of cancer treatment. Furthermore, delivery methods with high bioavailability (such as intravenous injection) should be employed in clinical trials involving ALA to effectively avoid a reduction in research due to high loss of and inability to effectively deliver ALA.

In conclusion, ALA is a promising anticancer molecule that exerts multilevel anticancer effects throughout the process of tumor prevention, occurrence, and treatment.
